# Bibliometric analysis of trends in research of *Tripterygium wilfordii* Hook F for treating rheumatoid arthritis

**DOI:** 10.1097/MD.0000000000036338

**Published:** 2023-11-24

**Authors:** Wenyuan Li, Chuanzhu Yan, Dongqing Du, Yuxia Ma

**Affiliations:** a Geriatric Medical Center, Affiliated Hospital of Shandong University of Traditional Chinese Medicine, Jinan, Shandong, China; b Prevention and Treatment Center, Affiliated Hospital of Shandong University of Traditional Chinese Medicine, Jinan, Shandong, China; c Department of Traditional Chinese Medicine External Treatment Center, Affiliated Hospital of Shandong University of Traditional Chinese Medicine, Jinan, Shandong, China; d Department of Acupuncture-Moxibustion and Tuina, Shandong University of Traditional Chinese Medicine, Jinan, Shandong, China.

**Keywords:** bibliometrics, Citespace, rheumatoid arthritis, *Tripterygium*

## Abstract

*Tripterygium wilfordii* Hook F (*Tw*HF) has been widely used to relieve rheumatoid arthritis (RA) in many countries. However, a bibliometric analysis of published articles discussing this treatment has not been conducted. This study aimed to explore the current status and trends of *Tw*HF for treating RA. Literature was extracted from the Science Citation Index Expanded Database of the Web of Science from January 1, 2013 to December 31, 2022. CiteSpace and the “bibliometrix” package were adopted to analyze the number of publications, countries, institutions, journals, authors, and keywords and to draw collaborative network maps. One hundred sixty-seven articles were identified. China has the most articles, followed by the United States. The China Academy of Chinese Medical Science had the study’s most significant publications and the highest centrality. The author analysis combined with the analysis of the cited authors, the rank of *Lin Na* is in an important position. The Journal of Ethnopharmacology, Frontiers in Pharmacology has published the most relevant articles and is the hottest related journal. For keyword analysis, “classification,” “criteria,” “mechanism,” and “methotrexate” were still being researched hot until 2022. Further investigation showed that “TNF-α,” “proliferation,” “endothelial growth factor,” “NF-κB,” and “collagen-induced arthritis” also remains research hotspot. Our results provide information on the research status, institutions, countries, authors, published journals, keywords related to using *Tw*HF to treat RA, and theoretical support for further research.

## 1. Introduction

Rheumatoid arthritis (RA) is a chronic inflammatory autoimmune disease characterized by joint pain, swelling, and stiffness.^[1]^ Genetics, female sex, and environmental factors are risk factors affecting the development of RA. Environmental factors include smoking, infectious agents, silica exposure, obesity, and changes in the microbiota.^[2]^ Uncontrolled RA causes joint damage, disability, and decreased quality of life.^[1]^

Nonsteroidal anti-inflammatory drugs, glucocorticoids, and disease-modifying antirheumatic drugs (DMARDs) (conventional DMARDs such as methotrexate, targeted DMARDs such as Janus kinase inhibitors, and biological DMARDs such as tumor necrosis factor-inhibitors)^[3]^ are effective for treating RA; however, their long-term use can lead to adverse consequences. Because of the insufficient response to these drugs and their related adverse events, novel drugs for treating RA are urgently needed.

*Tripterygium wilfordii* Hook F (*Tw*HF) is a traditional Chinese medicine with immunosuppressive effects that can activate blood circulation and collaterals, resist rheumatism, and relieve pain and swelling.^[4]^ *Tw*HF shows therapeutic effects for many diseases, such as Crohn disease and multiple autoimmune diseases.^[5–7]^ Moreover, *Tw*HF shows superior effects compared to methotrexate monotherapy and combination therapy with methotrexate and *Tw*HF in controlling disease activity in patients with active RA.^[8,9]^ The immunosuppressive, cartilage protective, and anti-inflammatory effects of *Tw*HF extract have been well demonstrated, and *Tw*HF extract is an alternative DMARDs suitable for traditional treatment of refractory RA patients.^[10]^ Further research on the mechanism of action of *Tw*HF in RA is necessary to enhance efficacy and reduce toxicity.

Bibliometrics is a discipline that applies quantitative methods such as mathematics and statistics to examine the distribution rules, quantitative relations of documents, and internal relations between documents based on the system and measurement characteristics.^[11]^ CiteSpace software can generate and analyze co-citation networks based on bibliographic records retrieved from the Web of Science.^[12]^ The Web of Science database used by Citespace is recognized as an authoritative citation document retrieval tool by the global academic community and is suitable for retrospective citation retrieval. Because of its academic nature and authority, the Web of Science database cannot index the many nontraditional forms of academic literature published online. Thus the comprehensiveness of its use for citation retrieval is limited.^[13]^

In this study, CiteSpace and R software were used to conduct bibliometric analysis including annual publications; most productive and influential countries, institutions, authors, journals; keywords to explore the current status, hotspots, and research trends of *Tw*HF for treating RA.

## 2. Materials and Methods

### 2.1. Data acquisition

**Figure 1.**
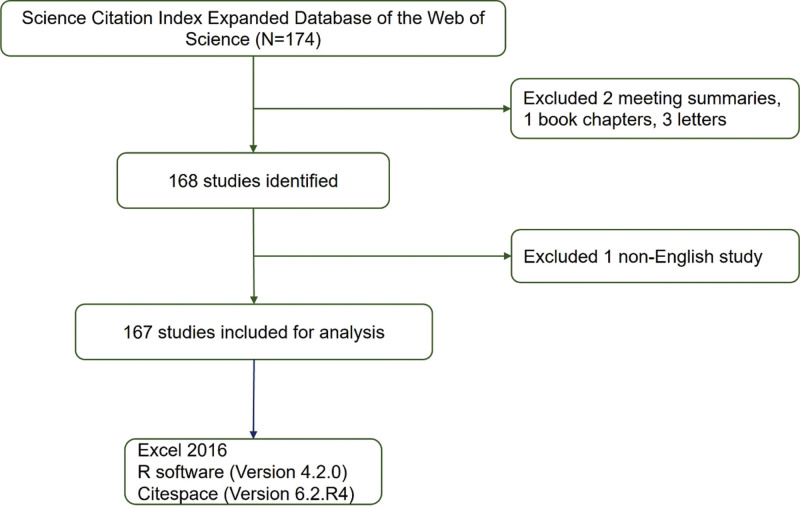
The flowchart of this study.

The data included in our study were screened from the Web of Science database from January 01, 2013 to December 31, 2022. The theme “rheumatoid arthritis” OR “RA” and “*Tripterygium wilfordii*” OR “*Tripterygium wilfordii* Hook F” were used as search terms. The document types were “article” and “review.” The document language is “English.” Repeated documents and those unrelated to the subject of the search were excluded (Fig. 1).

### 2.2. Analysis tool and parameter setting

Citespace 6.2.R4 (https://citespace.podia.com/), Excel2016, and R software (https://www.r-project.org/) were used for bibliometric analysis. The literature filtered from the Web of Science was saved in plain text and named download_**.txt. The Citespace software parameter settings included the following: Time slicing was from 2013 to 2022; Term source selected the Title, Abstract, Author Keywords (DE), Keywords Plus (ID); Term Type selected no terms; Links and Selection Criteria were used with default settings; and Pruning selected the Pathfinder and Pruning sliced networks.

## 3. Results

### 3.1. Annual publication

**Figure 2.**
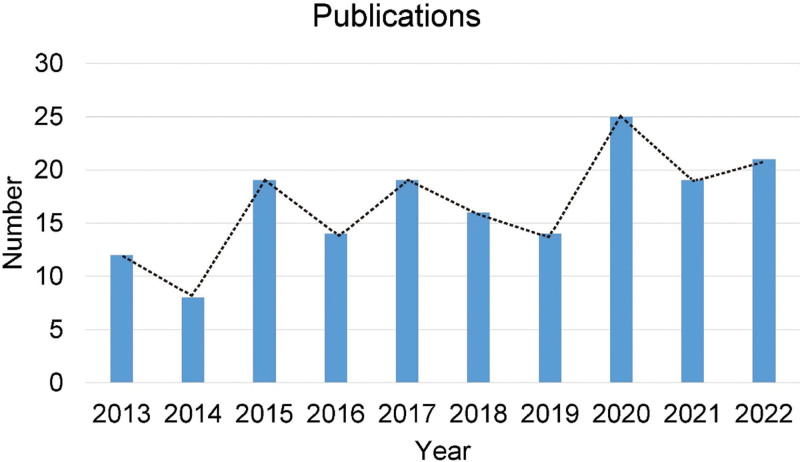
Number of publications from 2013 to 2022.

A total of 167 studies meeting the standards were screened from the Science Citation Index-Expanded Database of the Web of Science. Among them, there were 122 original research articles and 45 reviews. The number of annual publications fluctuated and increased over time (Fig. 2).

### 3.2. Most productive and influential countries

**Figure 3.**
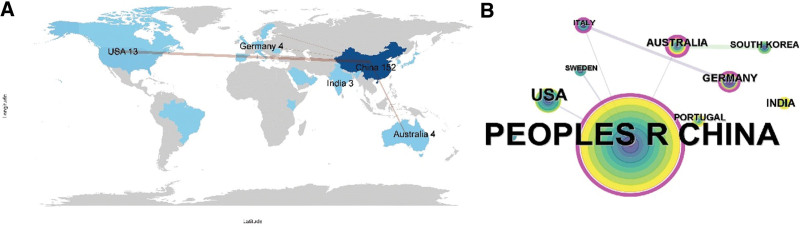
National/regional publication of *Tw*HF for treating RA. (A) The number of publications map, (B) the collaboration map. RA = rheumatoid arthritis, *Tw*HF = *Tripterygium wilfordii* Hook F.

Twenty-one countries have participated in the study of *Tw*HF for treating RA. R software and the “bibliometrix” package were used to plot the number and distribution of publications in different countries.^[14]^ China is the most productive country (n = 152), followed by the United States (n = 13), Australia (n = 4), and Germany (n = 4) (Fig. 3A). According to the countries collaboration analysis, China and the United States are the 2 most prominent connecting points (Fig. 3B).

### 3.3. Analysis of institutions

**Figure 4.**
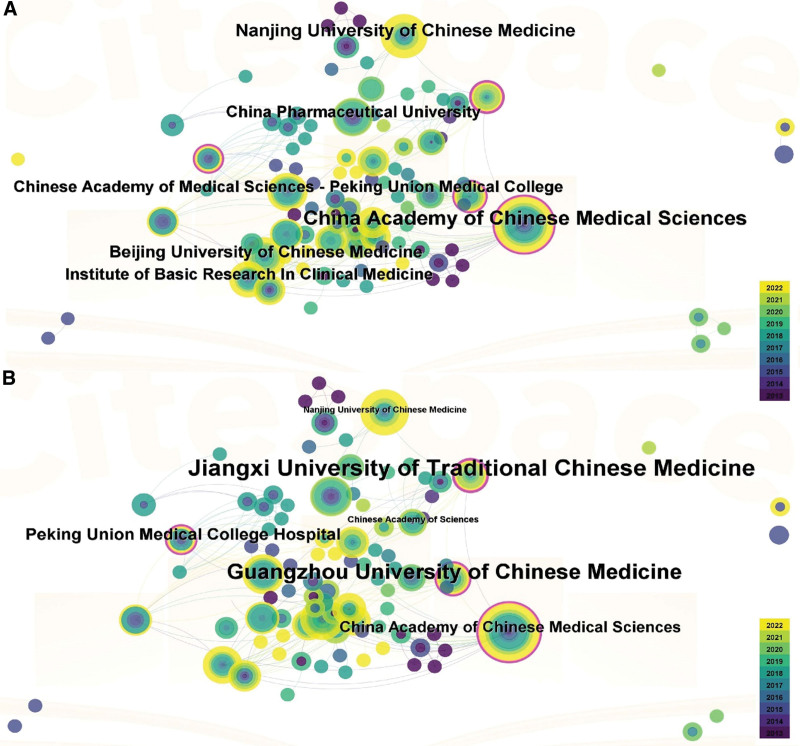
Institution map related to *Tw*HF for treating RA. (A) The number of institution publications map, (B) the institution collaboration map. RA = rheumatoid arthritis, *Tw*HF = *Tripterygium wilfordii* Hook F.

A total of 308 institutions participated in the study of *Tw*HF for treating RA. The top 5 institutions based on the number of publications were the Chinese Academy of Medical Sciences (n = 22), Nanjing University of Traditional Chinese Medicine (n = 14), Beijing University of Traditional Chinese Medicine (n = 12), China Pharmaceutical University (n = 11), Chinese Academy of Medical Sciences Peking Union Medical College (n = 11). Institute of Basic Research in Clinical Medicine CACMS (n = 11) (Fig. 4A). The top 5 institutions based on centrality were Jiangxi University of Traditional Chinese Medicine (Centrality = 0.21), Guangzhou University of Chinese Medicine (Centrality = 0.19), Peking Union Medical College Hospital (Centrality = 0.15), China Academy of Chinese Medical Sciences (Centrality = 0.14), Nanjing University of Chinese Medicine (Centrality = 0.08), Chinese Academy of Sciences (Centrality = 0.08) (Table S1, Supplemental Digital Content, http://links.lww.com/MD/K892, Fig. 4B).

The China Academy of Chinese Medical Science and the Nanjing University of Chinese Medicine ranked highest in the number of publications and centrality and are critical institutions at which studies of *Tw*HF for treating RA are being performed.

### 3.4. Analysis of authors

**Figure 5.**
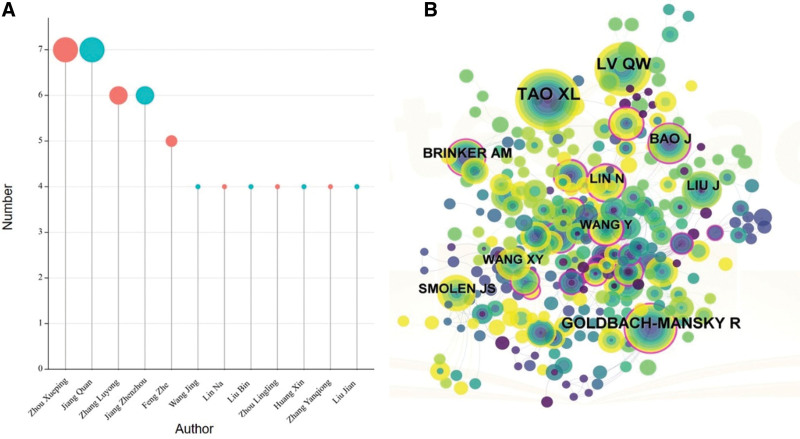
Author map related to *Tw*HF for treating RA. (A) The number of author publications map. (B) Cited authors map related to *Tw*HF for treating RA. RA = rheumatoid arthritis, *Tw*HF = *Tripterygium wilfordii* Hook F.

Based on the number of publications by the “bibliometrix” package, it was found that the top 4 authors were Zhou Xueping (n = 7), Jiang Quan (n = 7), Zhang Luyong (n = 6), Jiang Zhenzhou (n = 6), Lin Na (n = 4) (Fig. 5A). The cited author’s distribution map consisted of 299 nodes and 611 links. The top cited authors based on the cited counts were Tao Xuelian (n = 69), Lv Qianwen (n = 52), GOLDBACH-MANSKY R (n = 46), BRINKER AM (n = 27), Liu Jian (N = 24), Lin Na (n = 22) (Table S2, Supplemental Digital Content, http://links.lww.com/MD/K893, Fig. 5B). Lin Na ranked high in the number of publications and citation frequency.

### 3.5. Analysis of journals

**Figure 6.**
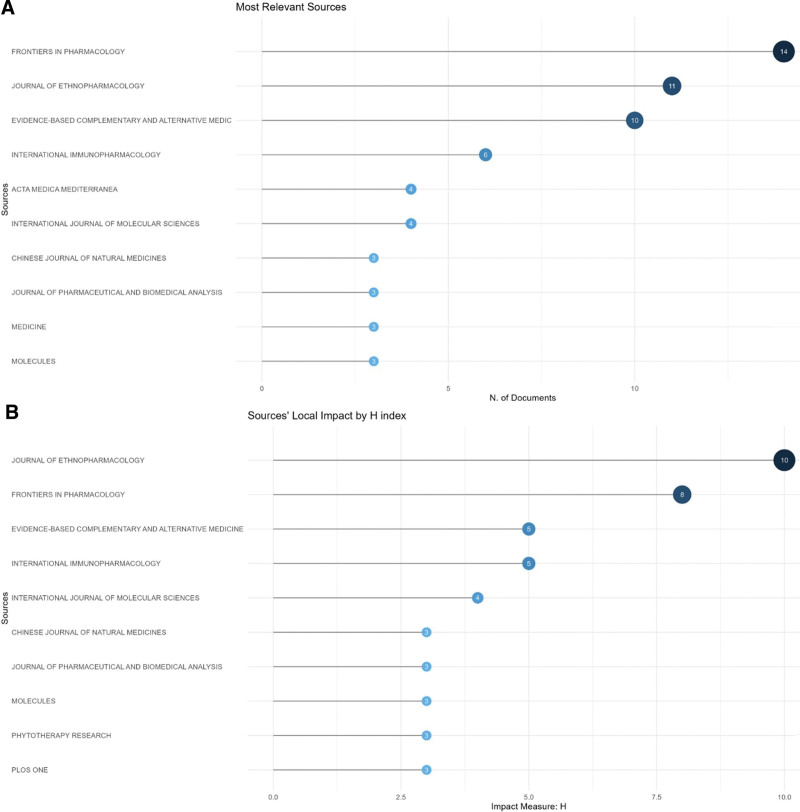
Journal map related to *Tw*HF for treating RA. (A) The number of journal publications map. (B) Journal impact map related to *Tw*HF for treating RA. RA = rheumatoid arthritis, *Tw*HF = *Tripterygium wilfordii* Hook F.

A total of 100 journals have published articles on *Tw*HF for treating RA. The top 5 journals by number of publications are Frontiers in Pharmacology (n = 14), Journal of Ethnopharmacology (n = 11), Evidence-based Complementary and Alternative Medicine (n = 10), International Immunopharmacology (n = 6), Acta Medica Mediterranea (n = 4) (Fig. 6A). According to Bradford law, based on the number of publications, Frontiers in Pharmacology, *Journal of Ethnopharmacology*, Evidence-based Complementary and Alternative Medicine, International Immunopharmacology, Acta Medica Mediterranea, *International Journal of Molecular Sciences, Chinese Journal of Natural Medicines, Journal of Pharmaceutical and Biomedical Analysis*, and Medicine are classified as core sources (Table S3, Supplemental Digital Content, http://links.lww.com/MD/K894).

With the biblioshiny, further analysis of journal influence was conducted. According to the H-index (threshold of 3, which meant each of them had at least 9 articles cited at least 9 times),^[15,16]^ the top journals were the Journal of Ethnopharmacology, Frontiers in Pharmacology, Evidence-based Complementary (Fig. 6B).

### 3.6. Analysis of keywords

**Figure 7.**
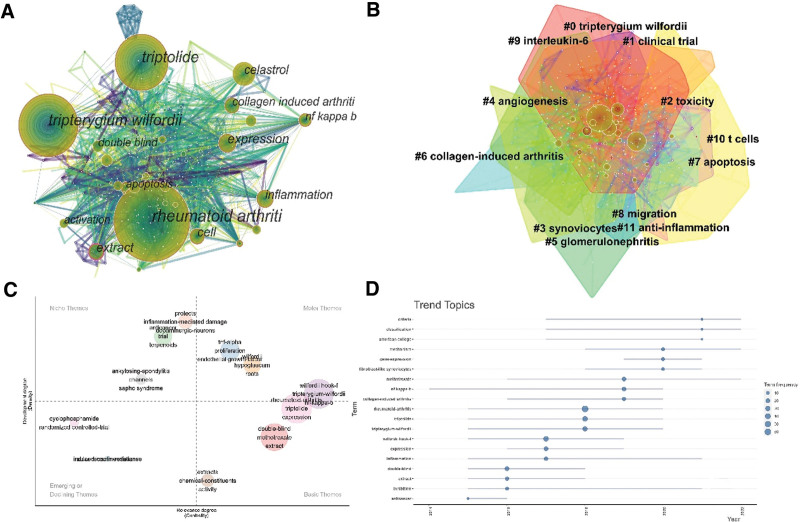
Keyword map related to *Tw*HF for treating RA. (A) Keywords co-appearance network map related to *Tw*HF for treating RA. (B) Keywords cluster map related to *Tw*HF for treating RA. (C) Thematic map analysis of keywords. (D) Trend topics analysis of keywords. RA = rheumatoid arthritis, *Tw*HF = *Tripterygium wilfordii* Hook F.

The distribution of the keyword co-appearance network map (Fig. 7A) consisted of 588 nodes and 2440 links. The top 5 keywords based on the number of publications were rheumatoid arthritis (215), *Tripterygium wilfordii* (141), triptolide (140), expression (59), and cell (52).

Based on keyword co-occurrence analysis, keywords cluster analysis was performed. The term extracted from the “keyword” was “cluster name,” and the cluster name was displayed using the “log-likelihood rate” algorithm. The modularity value, Q, represents whether the clustering network structure is significant, and the silhouette value, S, represents whether the clustering network result is credible; it is generally considered that Q > 0.3 indicates that the clustering structure is significant, S > 0.5 suggests that the clustering is reasonable, and S > 0.7 means that the clustering is convincing.^[12]^ The value of cluster Q was 0.3 ≥ 0.3, and that of cluster S was 0.8 > 0.7, indicating that the cluster structure is significant and the cluster is convincing. The clusters were #0 *Tripterygium wilfordii*, #1 clinical trial, #2 toxicity, #3 synoviocytes, #4 angiogenesis, #5 glomerulonephritis, #6 collagen-induced arthritis, #7 apoptosis, #8 migration, #9 interleukin-6 #10 T-cells, and #11 anti-inflammation (Fig. 7B). The “bibliometrix” package drew the thematic map to further explore the research hotspots and trends. The horizontal axis represents the centrality, and the vertical axis represents the density. The Motor Themes include “tnf-alpha”, “ proliferation”, “endothelial growth factor”, “nf-kappa-b”, “collagen-induced arthritis”, “ hypoglaucum”, and “ roots”. (Fig. 7C) Further, the trend topics of keywords analysis were carried out. The keywords “classification” and “criteria” have become increasingly popular since 2017 and peaked in 2021. The “mechanism” reached its hot peak in 2020, and “methotrexate” reached its hot peak in 2019. These 4 keywords were still being researched hot until 2022 (Fig. 7D).

## 4. Discussion

*Tw*HF is an effective and essential drug for treating RA. *Tw*HF-based therapies have been approved in China and are widely used to treat RA. Because of its clinical efficacy, *Tw*HF extract has also been evaluated worldwide in recent years.^[17]^ Research on the mechanism of *Tw*HF for treating RA has mainly focused on the molecular and genetic mechanisms. The molecular mechanisms mainly include; The influence on cytokines, particularly inflammatory cytokines, chemokines, and adhesion molecules, and their interactions;^[4,10,18]^ The influence on inflammatory mediators, mainly inhibiting the expression of cyclooxygenase and anti-oxidation, as well as reducing the production of nitrogen-free radicals;^[19,20]^ The impact on immune cells, mainly inhibiting the function of T-cells and secretion of proinflammatory factors by macrophages;^[21,22]^ The impact on cell signaling pathways, such as OPG/RANK/RANKL,^[23]^ the transcription factor NF-κB^[24]^ and signaling pathways, and; The influence on cell function, mainly cell proliferation and apoptosis. Studies of the epigenetic mechanisms mainly include; Histone modification, particularly the regulation of histone acetylation,^[25]^ and; miRNA, mainly the anti-inflammatory and protective effects of miRNA^[26]^ and the interaction with long noncoding RNA and mRNA.^[27]^

This study retrieved the literature on *Tw*HF for treating RA from the Web of Science. The author, institution, keyword, and other information in the literature were systematically mined and visualized using R software and Citespace software to intuitively display the literature publication trend, author cooperation and institution cooperation, main contents, and research hot spots in this field.

Analysis of the publications showed that China has the most significant number of publications. *Tw*HF is a traditional Chinese medicine, and its mechanism of activating blood circulation and collaterals and resisting rheumatism has attracted increasing interest in China.^[4]^ Analysis of publication trends showed that the number of publications fluctuated and improved each year. In 2013 to 2022, the number of publications on *Tw*HF for RA was small and considered a stable period. According to the number and trend of publications, treating RA with *Tw*HF remains the focus of research worldwide.

Institution analysis revealed that the China Academy of Chinese Medical Science and Nanjing University of Chinese Medicine are key institutions studying using *Tw*HF for treating RA. This research hotspot is the focus of various famous universities, with connections among institutions. All institutions should strengthen cooperation and joint research.

The author analysis combined with the study of the cited authors, the rank of Lin Na is in an important position. The review on *Tw*HF in the treatment of RA comprehensively expounds on the active components and mechanisms of *Tw*HF.^[4]^ The principal authors have formed their cooperative networks; however, there are few connections between them. Cooperation and information sharing should be strengthened among authors. In the study of *Tw*HF, the author should enhance domestic cooperation and strengthen international cooperation.

According to the analysis of the number and centrality of published literature, the journal Frontiers in Pharmacology (IF = 5.6) and Journal of Ethnopharmacology (IF = 5.4) ranked at the top. Studies of RA treatment with *Tw*HF have been published in well-known international journals.

In addition to RA and *Tw*HF, keywords such as expression and cells were essential in the keyword co-occurrence network. Thus, studies mainly involved target cells and related genes and cytokines of *Tw*HF during RA treatment.^[24,28–30]^ Based on keyword co-occurrence analysis, keyword clustering analysis was carried out. #3, #4, #6, #7, #8, #9, #10, and #11 are mainly studies of the mechanism of *Tw*HF for treating RA, and #2 and #5 are mainly studies of the side effects of *Tw*HF. “classification”, “criteria”, “mechanism”, and “methotrexate” were still being researched hot until 2022. Further analysis showed that “TNF-α”, “proliferation”, “endothelial growth factor”, “NF-κB”, and “collagen-induced arthritis” also remains research hotspot. Triptolide, an effective component of *Tw*HF, has been shown to reduce the levels of TNF-α, CXCL2, and VEGF in arthritis model. Meanwhile, triptolide inhibited the NF-κB signaling pathways, which in turn improved the RA joint inflammation and fixed immune imbalance.^[31]^

In summary, through our bibliometric analysis, we have made a visual summary of the research on *Tw*HF in RA, providing a reference for interested researchers. In terms of clinical research, the different therapeutic effects of *Tw*HF on various classification of RA can serve as a research direction. In terms of mechanism research, the classic pathway “NF-κB,” as well as TNF-α and VEGF, still have the potential to be further explored.

There were some limitations to our study. First, we only searched the Web of Science database, and thus the literature collection may not be comprehensive. Second, we used manual keyword combinations and screening for the collected literature; however, this did not affect the results of our study.

## Author contributions

**Conceptualization:** Wenyuan Li.

**Data curation:** Chuanzhu Yan, Dongqing Du.

**Investigation:** Chuanzhu Yan.

**Methodology:** Wenyuan Li.

**Software:** Wenyuan Li, Chuanzhu Yan.

**Supervision:** Yuxia Ma.

**Visualization:** Wenyuan Li.

**Writing – original draft:** Wenyuan Li.

**Writing – review & editing:** Dongqing Du, Yuxia Ma.

## Supplementary Material
