# Supplementary material for: Bibliometric analysis of trends in research of Tripterygium wilfordii Hook F for treating rheumatoid arthritis
Source: Medicine (Baltimore). 2023 Nov 24;102(47):e36338. doi: 10.1097/MD.0000000000036338 (PMC10681618; doi:10.1097/MD.0000000000036338)
Supplement: Supplementary file 1 [file medi-102-e36338-s001.docx]

**Table S1 Analysis of Institutions**

| Freq | Centrality | Label |
| --- | --- | --- |
| 7 | 0.21 | Jiangxi University of Traditional Chinese Medicine |
| 6 | 0.19 | Guangzhou University of Chinese Medicine |
| 5 | 0.15 | Peking Union Medical College Hospital |
| 22 | 0.14 | China Academy of Chinese Medical Sciences |
| 14 | 0.08 | Nanjing University of Chinese Medicine |
| 5 | 0.08 | Chinese Academy of Sciences |
| 2 | 0.07 | China Medical University Taiwan |
| 7 | 0.06 | Guang'anmen Hospital |
| 6 | 0.05 | China-Japan Friendship Hospital |
| 11 | 0.04 | Institute of Basic Research In Clinical Medicine |
| 7 | 0.04 | Hong Kong Baptist University |
| 6 | 0.04 | Peking University |
| 4 | 0.04 | Nanjing Medical University |
| 12 | 0.03 | Beijing University of Chinese Medicine |
| 5 | 0.03 | Institute of Chinese Materia Medica |
| 5 | 0.03 | Macau University of Science & Technology |
| 11 | 0.02 | Chinese Academy of Medical Sciences - Peking Union Medical College |
| 7 | 0.02 | Peking Union Medical College |
| 3 | 0.02 | Henan University of Traditional Chinese Medicine |
| 2 | 0.02 | Fujian University of Traditional Chinese Medicine |
| 2 | 0.02 | Army Medical University |
| 2 | 0.02 | National Institutes of Health (NIH) - USA |
| 2 | 0.02 | NIH National Institute of Arthritis & Musculoskeletal & Skin Diseases (NIAMS) |
| 11 | 0.01 | China Pharmaceutical University |
| 6 | 0.01 | Capital Medical University |
| 5 | 0.01 | Shanghai University of Traditional Chinese Medicine |
| 3 | 0.01 | Hebei Medical University |
| 2 | 0.01 | Academy of Military Medical Sciences - China |
| 7 | 0 | Anhui University of Chinese Medicine |
| 4 | 0 | Guangdong Pharmaceutical University |
