# Supplementary material for: Bibliometric analysis of trends in research of Tripterygium wilfordii Hook F for treating rheumatoid arthritis
Source: Medicine (Baltimore). 2023 Nov 24;102(47):e36338. doi: 10.1097/MD.0000000000036338 (PMC10681618; doi:10.1097/MD.0000000000036338)
Supplement: Supplementary file 2 [file medi-102-e36338-s002.docx]

**Table S2 Analysis of Cited Authors**

| Number | Degree | Centrality | Author |
| --- | --- | --- | --- |
| 69 | 4 | 0.05 | TAO XL |
| 52 | 1 | 0 | LV QW |
| 46 | 8 | 0.11 | GOLDBACH-MANSKY R |
| 29 | 6 | 0.15 | BAO J |
| 27 | 10 | 0.13 | BRINKER AM |
| 24 | 14 | 0.08 | LIU J |
| 23 | 9 | 0.09 | SMOLEN JS |
| 22 | 19 | 0.53 | LIN N |
| 20 | 23 | 0.3 | WANG Y |
| 20 | 6 | 0.06 | WANG XY |
| 18 | 11 | 0.13 | ALETAHA D |
| 18 | 4 | 0.03 | LI J |
| 17 | 11 | 0.33 | MA J |
| 16 | 8 | 0.15 | CIBERE J |
| 16 | 3 | 0.01 | LI XJY |
| 15 | 11 | 0.1 | CANTER PH |
| 15 | 6 | 0.09 | WANG X |
| 14 | 5 | 0.05 | ZHANG W |
| 13 | 19 | 0.14 | LIU QY |
| 13 | 6 | 0.12 | HAN R |
| 12 | 5 | 0.01 | LU Y |
| 12 | 2 | 0.01 | WANG J |
| 11 | 5 | 0.02 | ZHANG J |
| 11 | 2 | 0.01 | FAN DP |
| 10 | 4 | 0 | MCINNES IB |
