# Supplementary material for: Bibliometric analysis of trends in research of Tripterygium wilfordii Hook F for treating rheumatoid arthritis
Source: Medicine (Baltimore). 2023 Nov 24;102(47):e36338. doi: 10.1097/MD.0000000000036338 (PMC10681618; doi:10.1097/MD.0000000000036338)
Supplement: Supplementary file 3 [file medi-102-e36338-s003.docx]

**Table S3 According to Bradford's Law, the Analysis of Journals (Zone 1)**

| SO | Rank | Freq | Zone |
| --- | --- | --- | --- |
| FRONTIERS IN PHARMACOLOGY | 1 | 14 | Zone 1 |
| JOURNAL OF ETHNOPHARMACOLOGY | 2 | 11 | Zone 1 |
| EVIDENCE-BASED COMPLEMENTARY AND ALTERNATIVE MEDICINE | 3 | 10 | Zone 1 |
| INTERNATIONAL IMMUNOPHARMACOLOGY | 4 | 6 | Zone 1 |
| ACTA MEDICA MEDITERRANEA | 5 | 4 | Zone 1 |
| INTERNATIONAL JOURNAL OF MOLECULAR SCIENCES | 6 | 4 | Zone 1 |
| CHINESE JOURNAL OF NATURAL MEDICINES | 7 | 3 | Zone 1 |
| JOURNAL OF PHARMACEUTICAL AND BIOMEDICAL ANALYSIS | 8 | 3 | Zone 1 |
| MEDICINE | 9 | 3 | Zone 1 |
